# Hemolytic Disease of the Newborn Caused by Anti-Cellano (Anti-k) Alloimmunization: A Case Report

**DOI:** 10.7759/cureus.104540

**Published:** 2026-03-02

**Authors:** Marco A Paez, Derly Dallana Melo Ortiz, Sergio D. Cruz-Romero

**Affiliations:** 1 Pathology Department, Fundación Santa Fe de Bogotá, Bogotá, COL

**Keywords:** blood group, hemolytic disease of the fetus and newborn, hemolytic disease of the newborn, newborn blood transfusion, the kell blood group

## Abstract

This report describes a rare case of hemolytic disease of the newborn caused by anti-Cellano (anti-k) antibodies, part of the clinically significant Kell blood group system, the third most important blood group system after ABO and Rh. We present the case of a 43-year-old woman with a history of multiple miscarriages and one previous live birth conceived through in vitro fertilization with a donor oocyte. At 38 weeks of gestation, she delivered a B-positive neonate who subsequently developed anemia and jaundice. Laboratory evaluation demonstrated a positive direct Coombs test and the presence of anti-k antibodies in the mother, confirming the diagnosis. Initial management included intensive phototherapy, intravenous immunoglobulin, erythropoietin, and iron supplementation. Despite supportive therapy, hemoglobin levels progressively declined, and red blood cell transfusions became necessary. Because compatible k-negative donor units were not readily available, the mother, confirmed to be k-negative, donated blood for transfusion, resulting in clinical and hematologic improvement. This case highlights the importance of recognizing rare blood group incompatibilities and performing comprehensive immunohematologic evaluation in neonates with unexplained hemolysis.

## Introduction

The frequency of erythrocyte antigens within the Kell blood group system has been extensively characterized across different populations due to its significant immunohematological relevance. In the Maule region of Chile, the Kell (K, K1) antigen was found in 4% of blood donors, while the Cellano (k, K2) antigen was present in 99.5% of the population [1]. A similar study in eastern Colombia reported a 7.5% frequency for the Kell antigen among female blood donors [2].

The Kell system, considered the third most important blood group system after ABO and Rh, comprises more than 38 recognized antigens. Among these, K (KEL1) and k (KEL2, Cellano) are the most clinically significant due to their high immunogenicity. The Kell antigens are encoded by the KEL gene on chromosome 7q34 and produce a 732-amino-acid single-pass transmembrane glycoprotein, with a molecular weight of 93 kDa. This glycoprotein is expressed from early gestation and is present on mature erythrocytes, erythroid progenitors, myeloid cells, and other tissues such as bone marrow and testes, which may also have implications for solid organ transplantation [3,4].

The clinical significance of Kell alloimmunization was first recognized in 1949 by Dr. Levine, who described a case of mild hemolytic disease of the newborn (HDN) caused by maternal antibodies directed against the Cellano antigen [5]. Since then, Cellano-related hemolytic disease has been rarely reported, likely due to the high prevalence of the k antigen, absent in approximately 0.2% of individuals of Caucasian descent [6].

Despite its rarity, anti-Cellano (anti-k) alloimmunization can have serious clinical consequences. In sensitized mothers lacking the k antigen, the production of anti-k (IgG) antibodies may occur following pregnancy or transfusion, leading to the transplacental passage of maternal antibodies and immune-mediated destruction of fetal red blood cells. This can result in varying degrees of fetal anemia, hyperbilirubinemia, and even intrauterine fetal demise [7,8]. Moreover, these antibodies may also suppress fetal erythropoiesis, contributing to the severity of anemia, similar to the mechanism described for anti-K [9].

This report presents a rare but clinically relevant case of hemolytic disease of the newborn secondary to maternal anti-k alloimmunization, highlighting the importance of early identification and appropriate perinatal management.

## Case presentation

A 43-year-old woman was admitted to a tertiary hospital for a scheduled cesarean section at 38 weeks of gestation, following an uneventful prenatal care. This was her fifth pregnancy, achieved via in vitro fertilization with donor eggs. Her obstetric history included one live birth to the first pregnancy, followed by three miscarriages. Genetic testing performed after each miscarriage revealed fetal trisomy 17, a rare chromosomal abnormality typically incompatible with life. The patient had no prior history of blood transfusions and was blood typed as O-positive. The spouse and the first child were O-positive as well, although their Cellano (k antigen) status was not determined. The cesarean section proceeded without complications, delivering a newborn weighing 2680 g, appropriate for gestational age, with Apgar scores of 8, 9, and 10 at one, five, and 10 minutes, respectively. Antenatal fetal ultrasounds showed no signs of fetal anemia or fetomaternal hemorrhage. The neonate adapted well post-birth, with no signs of infection at the umbilical stump. However, initial oxygen saturation was 71%, indicating hypoxemia, which resolved with supplemental oxygen via a hood. This transient hypoxemia may have been related to delayed fetal lung fluid clearance or transient tachypnea of the newborn (TTN), both common in term infants delivered by cesarean section. Neonatology follow-up was advised.

**Figure 1:**
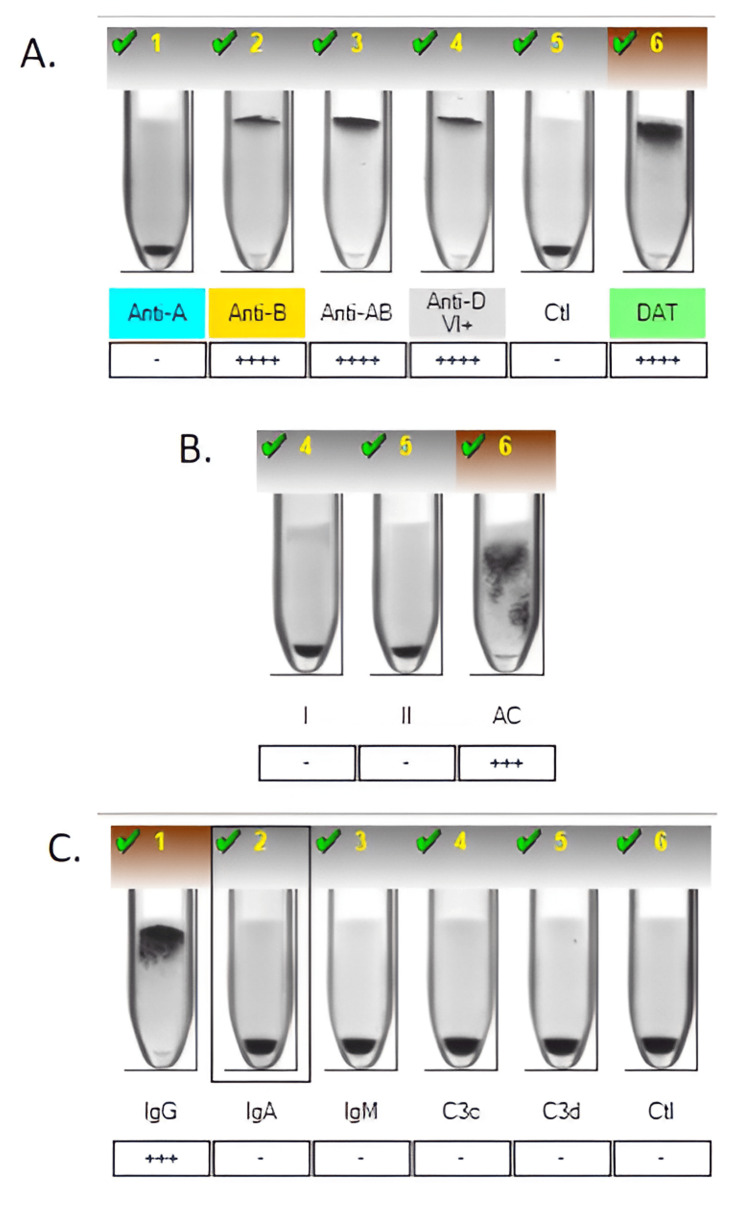
(A) Automated hemoclassification of the newborn showing RhD-positive blood group B. The direct Coombs test well shows a 4++++ positive reaction. (B) Screening for irregular antibodies in the newborn was negative, with a positive self-control (3+). (C) Direct Coombs fractionation demonstrating IgG positivity The images were obtained directly from the BANYO card reader analyzer, model 9940, serial number 2470, which captures and archives immunohematology test images through its integrated software system.

**Figure 2:**
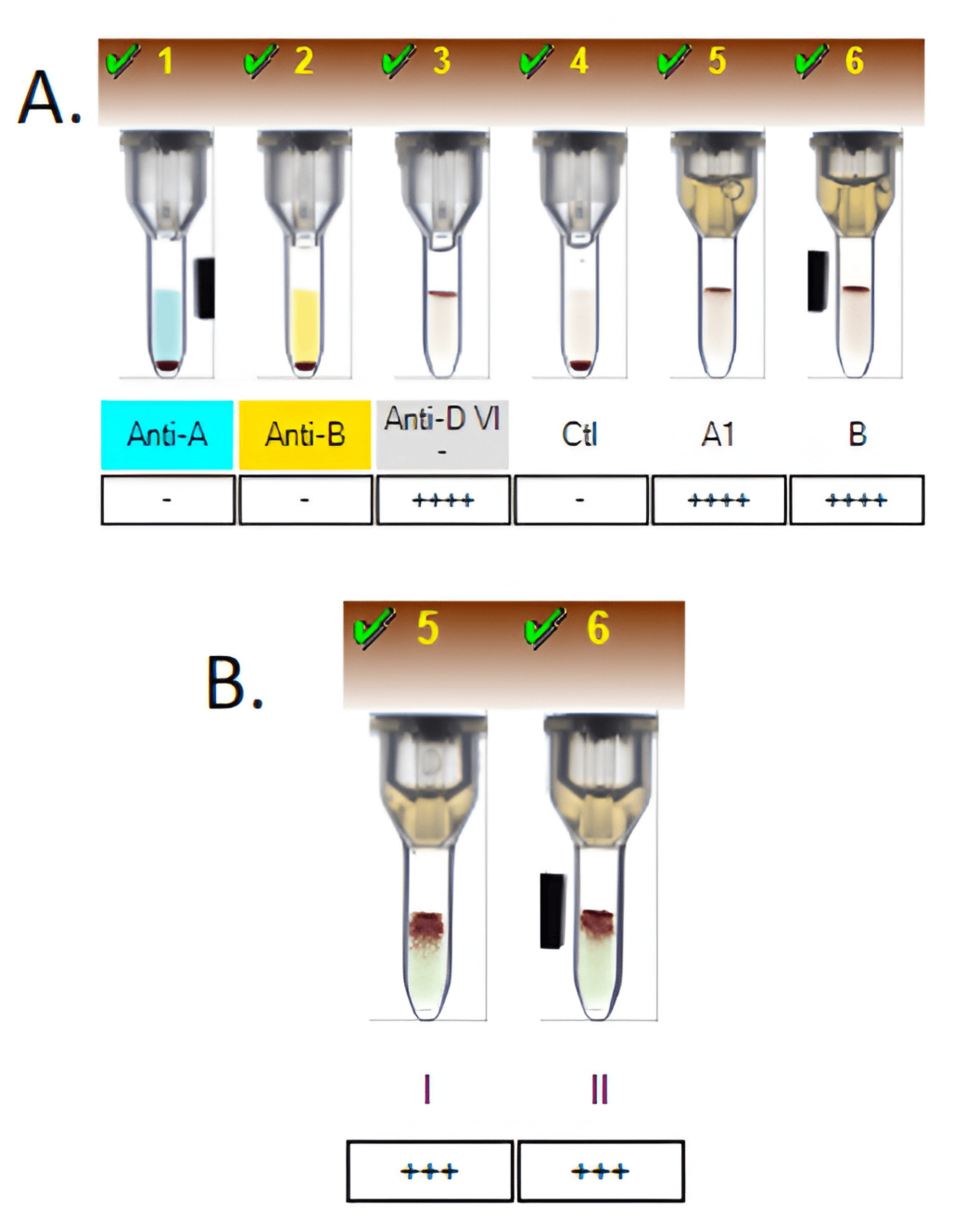
(A) Automated hemoclassification of the maternal blood sample showing RhD-positive blood group O. (B) Antibody screening demonstrating positive reactions with screening cells I and II. Duplicate testing using the same and a new sample confirmed persistent positivity with screening cells I and II. These screening cells correspond to a commercial panel distributed by Biocientífica The images were obtained directly from the BANYO card reader analyzer, model 9940, serial number 2470, which captures and archives immunohematology test images through its integrated software system.

Umbilical cord blood typing revealed the neonate to be B-positive, with a positive direct Coombs test (3+), which was performed due to clinical suspicion of hemolytic disease of the fetus and newborn (HDFN), as per institutional protocol (Figure 1). This prompted a notification to the neonatology team. Antibody screening of the mother showed a positive result, and further analysis identified the presence of anti-k antibodies, confirmed by a reference laboratory (Figure 2).

Subsequent neonatal investigations on day 2 of life revealed the following: transcutaneous bilirubin (BiliCheck) at 5.8 mg/dL, hemoglobin at 12.4 g/dL, hematocrit at 37.8%, neutrophils at 78.8%, lymphocytes at 12%, platelets at 463,000 µL, total bilirubin at 7.94 mg/dL, direct bilirubin at 0.76 mg/dL, and indirect bilirubin at 7.18 mg/dL. Reticulocyte count and serum lactate dehydrogenase (LDH) levels were not available in the medical records (Table 1). A diagnosis of early neonatal jaundice due to blood group incompatibility and anemia was established. Initial management in the neonatal ICU included intensive phototherapy and intravenous human immunoglobulin (5 g/100 mL, 1.34 grams every 24 hours). Subsequent treatment consisted of intravenous erythropoietin (2000 IU/0.3 mL, 1,080 IU every 24 hours) and oral ferrous sulfate drops (three drops every 12 hours).

**Table 1:** Laboratory results for the neonate

Parameter	Result	Reference range
Transcutaneous bilirubin (BiliCheck)	5.8 mg/dL	<5 mg/dL
Hemoglobin	12.4 g/dL	14-24 g/dL (neonates)
Hematocrit	37.8%	42-65% (neonates)
Neutrophils	78.8%	40-70%
Lymphocytes	12%	20-40%
Platelets	463,000 µL	150,000-450,000 µL
Total bilirubin	7.94 mg/dL	1-12 mg/dL (neonates)
Direct bilirubin	0.76 mg/dL	0-0.3 mg/dL
Indirect bilirubin	7.18 mg/dL	Calculated as total bilirubin-direct bilirubin
Hemoglobin (follow-up)	10.7 g/dL	14-24 g/dL (neonates)
Hematocrit (follow-up)	30%	42-65% (neonates)
Total bilirubin (follow-up)	10.38 mg/dL	1-12 mg/dL (neonates)
Indirect bilirubin (follow-up)	9 mg/dL	Not specified
Hemoglobin (post-transfusion)	14.9 g/dL	14-24 g/dL (neonates)
Hematocrit (post-transfusion)	44%	42-65% (neonates)
Total bilirubin (post-transfusion)	6.76 mg/dL	1-12 mg/dL (neonates)

Follow-up revealed a decrease in hemoglobin levels (10.7 g/dL) and hematocrit (30%), with raised bilirubin levels (total: 10.38 mg/dL; indirect: 9 mg/dL). Given the clinical status, a decision was made to proceed with red blood cell transfusion. The decision to transfuse was based on the progressive decline in hemoglobin and clinical assessment of ongoing hemolysis, according to institutional neonatal care protocols.

Due to the difficulty in sourcing Cellano-negative donors, the mother was considered the first option. After confirming her Cellano-negative status, she donated blood without complications. A 20 cc/kg dose of leukoreduced and irradiated red blood cells was transfused over three hours without any adverse events. Subsequent laboratory tests showed improvement, with hemoglobin rising to 14.9 g/dL and hematocrit to 44% and total bilirubin decreasing to 6.76 mg/dL. Following clinical stabilization, the neonate was discharged with outpatient oximetry and oral iron supplementation.

## Discussion

The incidence of HDFN or post-transfusion reactions due to anti-k antibodies is exceedingly rare, particularly because the k antigen has a very high prevalence (>99%) in most populations [3-5]. Nonetheless, the case presented here offers clinically relevant insights into the diagnosis, immunological basis, therapeutic approach, and prognosis in such scenarios.

Anti-k antibodies belong to the Kell blood group system and are highly immunogenic. When a mother lacks the k antigen and is exposed to it through fetomaternal hemorrhage, typically during a previous pregnancy, she may develop IgG antibodies that can cross the placenta in subsequent gestations [3,6,7]. In contrast to anti-D or ABO antibodies, anti-k antibodies not only cause hemolysis of circulating fetal erythrocytes but also suppress erythropoiesis by targeting erythroid precursors [8-10]. This dual mechanism can result in severe anemia, often out of proportion to bilirubin levels, and helps explain the need for red blood cell transfusion despite supportive therapies such as erythropoietin and intravenous immunoglobulin [8-11].

In this case, the mother had no history of transfusion, suggesting alloimmunization likely occurred during her first pregnancy, as has been described in similar clinical contexts [5,11]. Although the Cellano phenotype of the father was not tested, the newborn was confirmed Cellano-positive. This supports the hypothesis that paternal k antigen expression contributed to maternal sensitization and subsequent alloimmune complications. 

Interestingly, the mother's reproductive history included three first-trimester miscarriages following her first delivery. These were genetically characterized as trisomy 17, a rare and typically lethal chromosomal anomaly. Although these miscarriages occurred prior to the current pregnancy, the potential role of prior alloimmunization in exacerbating fetal demise, especially in the presence of chromosomal abnormalities, cannot be excluded. Literature suggests that Kell alloimmunization may impair erythropoiesis and placental function, potentially worsening adverse outcomes in genetically compromised fetuses [12,13].

Furthermore, in vitro fertilization with donor oocytes in this pregnancy introduced a different antigenic profile, possibly explaining the ABO incompatibility between the O-positive mother and the B-positive neonate. This immunologic mismatch highlights the complexity of managing pregnancies conceived through assisted reproductive technologies when maternal alloimmunization is present.

Fetal surveillance during this pregnancy did not show signs of anemia or hydrops on ultrasound, possibly due to late-onset immune activity or limited antibody titers during gestation. Nonetheless, postnatal findings included a positive direct Coombs test and early-onset jaundice, with laboratory evidence of hemolysis and anemia. Although universal screening for direct Coombs test is not routine in some settings, it was clinically indicated in this case due to the constellation of anemia, hyperbilirubinemia, and maternal history [10,14].

Initial management included intensive phototherapy, intravenous immunoglobulin, and erythropoietin. The partial response to these therapies, evidenced by continued anemia and elevated bilirubin levels, necessitated red blood cell transfusion. Due to the scarcity of Cellano-negative donors, maternal blood was used after confirming her antigen-negative status. The transfusion was well tolerated and led to hematologic and clinical improvement, highlighting the importance of considering maternal donation when compatible units are difficult to obtain [15].

This case aligns with prior reports indicating that anti-k antibodies can cause severe neonatal anemia requiring transfusion, even in the absence of antenatal signs of HDFN [9,11,16]. It emphasizes the need for comprehensive postnatal monitoring in neonates with positive direct Coombs test or unexplained jaundice, even when antenatal imaging appears normal. The possibility of suppressed erythropoiesis should be considered in cases of persistent anemia despite supportive treatment [8,13].

While this case reinforces current understanding, it also raises considerations for future practice. The absence of prenatal ultrasound findings suggests that Kell-related HDFN may be underestimated when relying solely on imaging. Moreover, the complexity introduced by oocyte donation may require additional immunohematologic screening in assisted reproduction [14,17]. Molecular diagnostic tools, such as cell-free fetal DNA analysis for Kell genotyping, could enable earlier risk stratification and tailored monitoring in sensitized pregnancies [18].

## Conclusions

Anti-k alloimmunization represents a rare but clinically significant cause of hemolytic disease of the newborn. Early recognition through maternal antibody screening and neonatal direct antiglobulin testing enabled timely diagnosis and appropriate management in this case. The favorable outcome achieved through combined supportive therapy and targeted transfusion underscores the importance of individualized immunohematologic assessment in neonates with unexplained anemia and hyperbilirubinemia.
